# Near-Infrared Surface-Enhanced Raman Scattering on Silver-Coated Porous Silicon Photonic Crystals

**DOI:** 10.3390/nano9030421

**Published:** 2019-03-12

**Authors:** Marko Škrabić, Marin Kosović, Marijan Gotić, Lara Mikac, Mile Ivanda, Ozren Gamulin

**Affiliations:** 1 Department of Physics and Biophysics, School of Medicine, University of Zagreb, Šalata 3b, 10000 Zagreb, Croatia; marko.skrabic@mef.hr; 2 Research Unit New Functional Materials, Center of Excellence for Advanced Materials and Sensing Devices, Bijenička cesta 54, 10000 Zagreb, Croatia; marijan.gotic@irb.hr (M.G.); lara.mikac@irb.hr (L.M.); 3 Faculty of Science, University of Split, Ruđera Boškovića 33, 21000 Split, Croatia; marin.kosovic@pmfst.hr; 4 Laboratory for Molecular Physics, Division of Materials Physics, Ruđer Bošković Institute, Bijenička cesta 54, 10000 Zagreb, Croatia

**Keywords:** SERS, near-infrared, crystal silicon photoluminescence, porous silicon photonic crystals, hot-spots

## Abstract

Surface-enhanced Raman scattering (SERS) with near-infrared (NIR) excitation offers a safe way for the detection and study of fragile biomolecules. In this work, we present the possibility of using silver-coated porous silicon photonic crystals as SERS substrates for near-infrared (1064 nm) excitation. Due to the deep penetration of NIR light inside silicon, the fabrication of photonic crystals was necessary to quench the band gap photoluminescence of silicon crystal, which acts as mechanical support for the porous layer. Optimal parameters of the immersion plating process that gave maximum enhancement were found and the activity of SERS substrates was tested using rhodamine 6G and crystal violet dye molecules, yielding significant SERS enhancement for off-resonant conditions. To our knowledge, this is the first time that the 1064 nm NIR laser excitation is used for obtaining the SERS effect on porous silicon as a substrate.

## 1. Introduction 

Surface-enhanced Raman scattering (SERS) is an exceptionally powerful vibrational spectroscopy technique, which finds wide applications in the identification and structural studies of biological materials and chemical substances [1,2,3]. It has attracted a great deal of scientific interest due to a huge enhancement of the Raman signal from a small number of molecules near or bound to plasmonic surfaces, ultimately reaching single-molecule detection [4]. It is generally accepted that such a signal enhancement is attributed to the superposition of the two effects, the electromagnetic (EM) and the chemical enhancement mechanisms. The EM mechanism is dominant and originates from amplification of the EM fields on or in the immediate vicinity of roughened noble-metal surfaces generated by the excitation of localized surface plasmons, while the chemical mechanism results from charge-transfer between adsorbed molecules and the metal substrate, which increases the polarizability of molecules and thereby their Raman scattering cross-section [5]. 

Among the various promising SERS-active substrates [6], it has been shown that non-expensive, easy-to-fabricate, stable in air, uniform, reproducible, and highly sensitive SERS-active substrates can be produced using noble-metal nanoparticles deposited or grown on the porous silicon surface [7,8,9,10,11,12,13]. Recently, a thorough and perspicuous review about SERS on metal-coated porous silicon as a substrate has also been published [14].

Porous silicon (pSi), most commonly obtained on top of a crystalline silicon (cSi) wafer by its anodization in hydrofluoric acid (HF) solution, is a versatile nanostructured material known for its many unique optical, chemical, and physical properties and its corresponding usage [15]. By variation of the anodization parameters, porous layers with tunable pore sizes and thicknesses can be produced [16]. Moreover, the obtained vast active surface area has an inherent property to spontaneously reduce metallic ions, which have positive reduction potentials with respect to hydrogen when immersed in their aqueous solutions [17,18,19]. Utilizing this feature, immersion plating has become the most common method to coat pSi with certain metals (Ag, Au, Cu) and form SERS-active substrates due to its simplicity, low cost, and, most importantly, control of the substrate morphology by a precise variation of the deposition conditions [20,21,22].

So far, SERS measurements on pSi were conducted with excitation wavelengths in the visible spectral range, where the strongest Raman enhancement was expected due to the matching of the laser wavelength with the localized surface plasmon resonance of the metal/pSi substrates [23]. Extending the wavelength range to the near-infrared (NIR) at 1064 nm, despite the loss of sensitivity due to the Raman’s scattering fourth-power dependence on the excitation frequency, has advantages in the absence of resonance-Raman effects for the majority of molecules, the reduction of fluorescent photobleaching and plasmonic heating, as well as the avoidance of possible photodegradation of biological molecules [24,25,26].

Although NIR SERS with an excitation at 1064 nm has been demonstrated to be operative during the past 30 years [24,27,28,29,30], hitherto, metal-coated pSi was not used as an SERS-active substrate for NIR excitation. The main reason is probably the deep penetration of NIR light inside cSi or pSi [31,32], hence such an excitation induces the photoluminescence (PL) of the underlying cSi [33,34] due to the energy matching with the cSi band gap, and consequently the SERS signal is concealed with the broad PL peak. On the other hand, the usage of metal-coated free-standing pSi as the SERS substrate, obtained by the detachment of the porous layer from the underlying cSi wafer and subsequent metallization, although possible, does not meet standard SERS requirements for a robust substrate because of its pronouncedly fragile nature. 

More than 20 years ago, pSi multilayers were produced [35,36] by the utilization of another of its important properties; the fact that already etched porous layers are not affected during the electrochemical anodization, i.e., cSi dissolution occurs only at the etching front, which is the interface between pSi and cSi. Thus, by varying the current density applied during the etch process, the porosity can be modulated in the direction perpendicular to the pSi surface and, consequently, almost any refractive index-depth profile can be realized. This allows the fabrication of a variety of pSi structures with desired optical properties and with a wide range of applications, such as omnidirectional mirrors [37], chemical and biological sensors [38,39], waveguides [40], and biomolecular screening [41]. Among those, periodic structures that can control the propagation of a certain frequency range of light are called porous silicon photonic crystals (pSi PhC) [42]. They are characterized by a high reflectivity stopband, which can be tuned to appear anywhere in the predetermined spectral region depending on the appropriate selection of fabrication parameters. 

The aim of this study is the structural optimization of SERS-active silver-coated pSi substrate for NIR excitation. To obtain the SERS effect, pSi PhC with efficient reflectance in the NIR spectral range (~1064 nm) that quenches the cSi substrate band gap PL had to be produced. Here, we report a detailed fabrication of porous silicon rugate filters (pSi RFs) and subsequent synthesis of nanostructured silver (Ag) coating with appropriate morphology by immersion plating of pSi RFs in Ag salt aqueous solution. The SERS ability of such substrates was evaluated using aqueous/ethanolic solutions of rhodamine 6G (R6G) and crystal violet (CV) dyes at various concentrations. 

## 2. Materials and Methods

The cSi wafers used in this study were p-type, Boron doped, with a resistivity of 0.001–0.002 Ohm cm, (100) crystal face orientation, and 525 ± 25 µm thickness, obtained from Prime Wafers Inc. Prior to anodization, the cSi wafers were cut into ~4 cm^2^ squares and rinsed in acetone. The etching process was performed in a home-made Teflon electrochemical etch cell with an aluminium foil as a back contact and platinum mesh, suspended on a fixed height above the polished side of the cSi wafer, as a counter electrode. The exposed surface of the cSi wafer electrode was approximately 2.5 cm^2^, adjusted by an O-ring at the bottom of the cell. All pSi samples were obtained using a LabVIEW-controlled (National Instruments, Austin, TX, USA) current source (2601B SourceMeter, Kiethley Instruments Inc., Solon, OH, USA) in a solution composed of 40 wt%. HF (Sigma Aldrich, Taufkirchen, Germany) and 99% v/v ethanol in a volume ratio of 3:1. 

To obtain reproducible surfaces prior to fabricating pSi PhC, a precleaning procedure on the pSi wafers was performed using a constant current density of 200 mA/cm^2^ for 30 s, followed by dissolving the sacrificial porous layer in 1 M KOH solution [16]. For pSi RFs, the current density was modulated with a sinusoidal waveform oscillating between 1 and 100 mA/cm^2^ and repeated for 40 or 80 cycles. The periods were between 4.6 and 4.9 s and these conditions yielded a mesoporous structure with 64% porosity. For ordinary one-layer pSi samples, the anodization process was carried out using a constant current density of 50.5 mA/cm^2^ with 200 s etching duration. After etching, the samples were rinsed twice in ethanol and dried under a gentle stream of nitrogen gas. Table 1 summarizes the characteristics of the samples used in this study. The porosity of the samples was determined gravimetrically by measuring the sample masses on a laboratory balance with a resolution of 10 µg. The thus prepared pSi and pSi RF samples remained attached to the cSi wafer substrate throughout the rest of the study.

Just before the immersion plating procedure, the pSi and pSi RF samples were mechanically divided into small rectangles with a surface area of about 1–2 mm^2^ and dipped for 2 min in a 2 wt% HF solution to remove the native oxides from the surface, followed by rinsing with ethanol. Ag nanostructures were deposited on the top surface of a porous layer by immersing the prepared pSi and pSi RF samples into 10^−2^ M AgNO_3_ aqueous solution for 1 to 35 min. The thus prepared SERS-active substrates were rinsed in water and left to dry in air. When necessary, the Ag-coated pSi and pSi RF samples were immersed into a 10 mM HCl solution for 10 s to remove any contaminant adsorption on a high surface area of Ag-coated pSi and pSi RF samples during the specimen preparation and storage.

As testing molecules for SERS R6G and CV were used; the stock 10^−2^ M analyte solutions were prepared by dissolving the solid powder in 1:1 water/ethanol volume ratio. Different concentrations (10^−3^ M to 10^−8^ M) were obtained by successive dilution of stock solutions. Ag-coated pSi and pSi RF samples were incubated for 30 min [43] in 200 μL of the analyte solutions to realize molecule adsorption. After incubation, samples were thoroughly rinsed with water and left to dry in air.

Raman and SERS measurements were performed in backscattering geometry using a continuous-wave Nd:YAG 1064 nm laser (9398 cm^−1^) as an excitation source with a PerkinElmer GX FT-Raman spectrometer. The spectra were recorded by averaging 10–100 scans in the 3500–200 cm^−1^ range with a spectral resolution of 4 cm^−1^. The laser power used was between 250–500 mW to avoid the influence of possible heating while the spot size on the samples was about 0.25 mm in diameter. 

The reflectance spectra of pSi PhC were measured by a UV–Vis–NIR spectrophotometer (Shimadzu UV-3600, Shimadzu Corp., Kyoto, Japan) with the integrated sphere in the total reflectance mode over the spectral range of 300–2400 nm with a resolution of 1 nm and a normal angle of incidence.

The structural properties of samples were determined using a JEOL JSM-7000F (Jeol Ltd., Tokyo, Japan) thermal field emission scanning electron microscopy (FE-SEM) operating at 3 and 5 kV accelerating voltage and with a working distance of ~10 mm.

All experimental procedures were done at room temperature. Millipore ultrapure water (Milli-Q, 18.2 MΩ/cm) was exclusively used.

## 3. Results and Discussion

### 3.1. Crystal Silicon Photoluminescence

**Figure 1:**
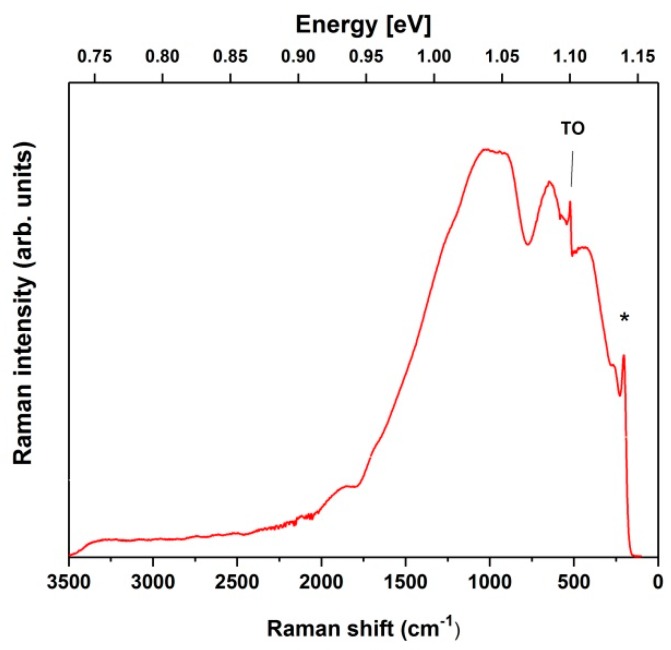
FT-Raman spectrum of pSi on top of highly doped cSi substrate. The band at ~520 cm^−1^ due to the transverse optical phonons (TO) is shown. The band marked with an asterisk represents instrumental artifact.

During the etch process, pSi is fabricated on top of the underlying cSi wafer, which mainly acts as mechanical support for the porous layer. The penetration depth of the near-infrared 1064 nm laser excitation used in this work, contrary to the visible lasers commonly used in SERS applications, is rather deep for silicon because of its low absorption coefficient at this wavelength [31], and therefore NIR light penetrates through the pSi into the cSi substrate. Since this excitation has a photon energy of ~1.165 eV, which is just slightly above the band gap of cSi (~1.115 eV at room temperature), strong cSi band gap PL is created due to radiative recombination of photogenerated charge carriers [44,45]. The pSi band gap is much wider [15], hence NIR light cannot induce its PL. Figure 1 shows the typical FT-Raman spectrum of pSi on top of the highly doped cSi substrate recorded with the near-infrared 1064 nm excitation. Superimposed on the broad PL background, only one sharp peak, located at ~520 cm^−1^ relative to the excitation line and corresponding to the Raman scattering by transverse optical (TO) phonons at the center of the Brillouin zone of cSi is clearly distinguishable. Other visible, much broader peaks and a tailing PL emission have been associated with dislocation effects related to strain, impurities, or ground boundaries in the cSi [33,45]. Due to this broad PL band, it is not convenient to use such spectrum as a referent one for SERS measurements.

To avoid the influence of the cSi substrate band gap PL, the porous layer can be detached from the cSi substrate during the final stages of the electrochemical etching process. Unfortunately, the thin films of free-standing pSi are extremely delicate to handle and are unfavourable for practical applications. Moreover, pSi samples with cSi bases can be easily diced to obtain the desired surface area for certain applications. Furthermore, cSi bases are important for possible technological applications, such as the integration of an SERS-active substrate with other elements on a single cSi wafer [15].

On the other hand, an additional possibility to eliminate undesirable cSi PL exists. As already briefly mentioned in the introduction section, the easiest and most common way to deposit Ag on pSi and thereby create SERS-active substrate is the immersion plating method. The spontaneous formation of Ag nanostructures on the surface of a pSi template during its immersion in AgNO_3_ solution proceeds in accordance to the Volmer-Weber mechanism and the model proposed by Harraz et al. [18]. The oxidation of the pSi surface occurs simultaneously with the Ag^+^ ion reduction and the whole deposition process continues by Ag island nucleation and growth, resulting in an oxidized pSi layer coated with Ag nanoparticles (Ag NPs). Generally, it is known that the deposition conditions have a determinable effect on the properties of the SERS-active substrates. For instance, Saito et al. [46,47] used the silver mirror reaction to produce silver films on silicon and transparent borosilicate glass. The size of the silver colloidal particles in these films could be tightly controlled by varying the reaction conditions. In the present contribution, the parameters of the immersion procedure, along with the pore diameters, interpore distance, and the thickness of the spongiform pSi surface, have a crucial influence on the morphology (average size, density, and arrangement) of the uniformly deposited Ag NPs and hence on the Raman signal enhancement of the adsorbed molecules [8,20,21,22,43]. After fixing the parameters, such as the AgNO_3_ concentration and temperature, increasing the time of deposition leads to an increase of Ag NPs’ size, aggregation of the Ag NPs to nanoclusters, and eventually nanocluster coalescence into a uniform layer. However, the deposition of metals is usually accompanied by the quenching of PL due to a fundamental disruption of the luminescence mechanism, increasing with the metal content in the layer [17,48], meaning that Ag crystals attract excited electrons, eliminating the possibility of recombination with holes. Moreover, metals are known for their high reflectivity in the NIR spectral region [49], hence prolonged Ag deposition leads to the formation of layers with efficient reflectance of the incoming 1064 nm laser excitation, thus preventing the cSi PL emergence.

**Figure 2:**
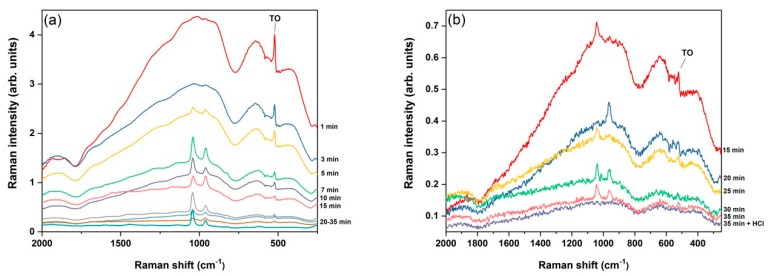
Comparison of FT-Raman spectra of pSi on top of a highly doped cSi substrate immersed in 10^−2^ AgNO_3_ for different deposition times: (**a**) 1–35 min (50 scans), (**b**) 15–35 min (10 scans).

Considering this, the pSi samples S1 were immersed in 10^−2^ M AgNO_3_ for different deposition times (1–35 min). With the increase of the deposition time, a gradual color change of the pSi surface from light grey to shiny metallic was observed, indicating the formation of a thick uniform Ag layer. The Raman spectra for this sequence of Ag/pSi samples are shown in Figure 2a. It is visible how cSi luminescence quenches with a longer exposure of samples to the Ag salt. To precisely determine the immersion time of the complete cSi PL disappearance, another set of Ag/pSi samples’ Raman spectra was recorded and is shown in Figure 2b with an expanded vertical axis, revealing that more than 30 min of dipping is needed.

Additionally, it can be seen that the TO cSi peak at ~520 cm^−1^ decreases at the prolonged immersion times and disappears after ~30 min, which is direct evidence that laser excitation does not penetrate into the cSi substrate. Two sharp peaks (that are possibly unreacted AgNO_3_ or Si-O vibrations) appearing at ~960 and ~1042 cm^−1^ after 5 to 7 min of immersion were successfully removed by short dipping in a diluted hydrochloric acid solution. This cleaning process had no influence on the FT-Raman spectrum of Ag-coated pSi, as can also be seen in Figure 2b.

**Figure 3:**
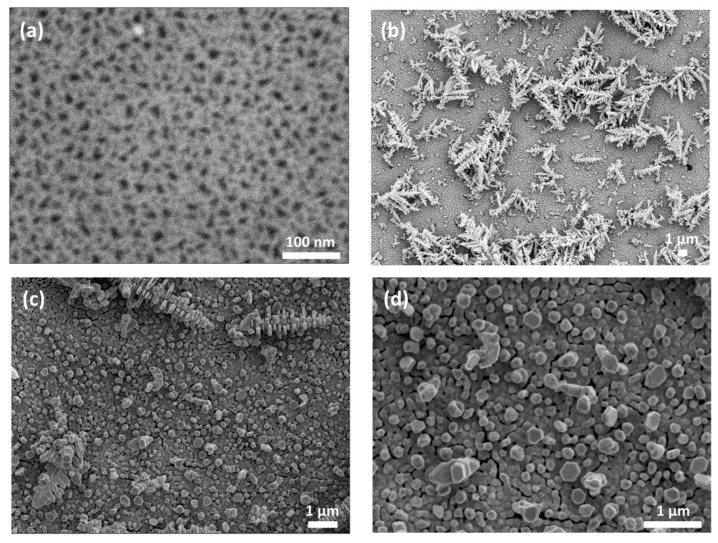
FE-SEM top view images of pSi S1 (**a**) mesoporous substrate prior to immersion in Ag salt; (**b**–**d**) micrographs of Ag particles formed on top of pSi by immersion plating for 30 min in 10^−2^ M AgNO_3_ at three magnifications.

The morphology of the pSi surface and Ag NPs immobilized on it was examined by FE-SEM. Figure 3a shows the top view FE-SEM image of pSi before immersion into Ag salt and reveals a dense mesoporous morphology with estimated average pore dimensions of ~10 nm. Figure 3b–d show the same sample after 30-min immersion into 10^−2^ M AgNO_3_ at different magnifications. It is visible that micrometer-sized Ag dendrites on top of the quasi-continuous Ag film cover the entire porous surface. At higher magnifications, it is evident that film formation consists of almost coalesced particles.

### 3.2. Porous Silicon Photonic Crystals

In this work, pSi PhC are utilized to block the penetration of 1064 nm NIR light into the cSi and hence inhibit the occurrence of the undesired cSi PL. During the electrochemical etching, the refractive index of pSi, being itself a function of the material’s porosity, is determined by the applied current density. A special class of pSi PhC, which has a continuous sinusoidal refractive index variation in the direction normal to the surface, iscalled pSi rugate filter (pSi RF) [50]. Compared to commonly used Bragg reflectors, which possess a slightly higher and wider reflectivity peak, usually called a stopband, pSi RF’s individual layers are not required to be phase-matched, hence they are much simpler to fabricate. The spectral position of this reflectivity peak, which arises from the multiple reflections and interference in the periodic internal structure, is described by the equation:

λ = 2*n_av_d*, (1)

where *n_av_* and *d* are the average effective refractive index of the porous structure and the period of one sinusoidal oscillation, respectively. Thus, for a fixed difference of anodization currents, the reflectivity peak shifts toward higher wavelengths by increasing the spatial thickness of each layer, which is proportional to the period of an etch cycle [51]. The width of this resonance feature, sometimes also called the photonic band gap (PBG), depends solely on the refractive index contrast and for that reason, we used highly doped cSi, whose porosity of the single layers can be tailored in a broader range compared to the less doped p-type cSi [36].

**Figure 4:**
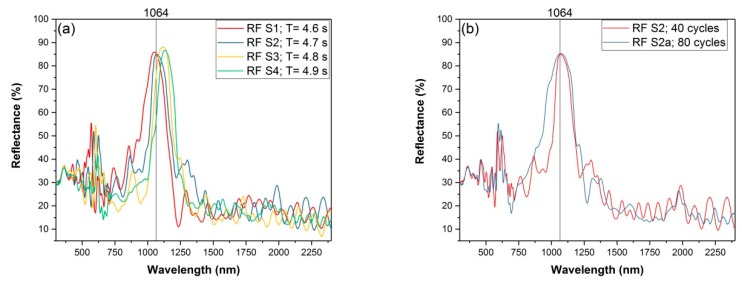
Reflectance spectra of pSi RFs (**a**) pSi RFs prepared using same sinusoidal current densities and same number of different periods (**b**) pSi RFs prepared with same period but different number of cycles. The vertical lines represent the wavelength of FT-Raman excitation (1064 nm).

Figure 4a shows the reflectance spectra of four pSi RFs prepared with the same etching conditions except slightly different sine periods. For fixed 40 sine cycles, the principal rugate stopband of the samples shifts from 1053 to 1132 nm as the period increases from 4.6 to 4.9 s. The reflectance is maintained at ~85% and the full width at half maximum of the reflection peaks vary between 160 and 200 nm. Since we only considered the performance within the stopband, deviations from the ideal pSi RF structure were of no concern. For instance, higher-order harmonics with smaller amplitudes, visible approximately at integer multiples of the energy of the first stopband, can otherwise be suppressed by creating rugate filters with a sinusoidal modulation of the logarithm of the refractive index, *n*, with depth [52] Also, no apodization or index matching functions [53] were applied to the refractive index profiles to reduce interference oscillations (Fabry-Perot fringes) appearing on the higher-wavelength side of the stopband and arising from reflections at the air/pSi and pSi/cSi interfaces. Although increasing the number of sinusoid etch repetitions for a given fixed period reduces the width and increases the reflectivity of a principal band up to a certain limit [54], we observed different behaviour, as seen in Figure 4b. This is probably because of depth inhomogeneities of the optical thickness of single layers, caused by the conductivity and local changes of the HF concentration [55,56], which smears out the principal band for longer etching times [36]. 

**Figure 5:**
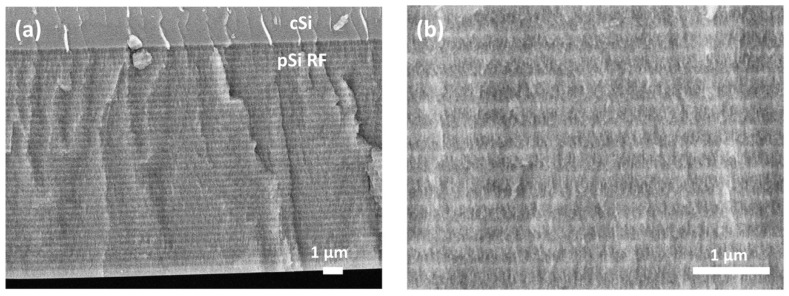
Cross-section FE-SEM images of an RF S2 consisting of 40 cycles recorded with two magnifications. (**a**) Low magnification image showing the whole rugate filter structure (**b**) High magnificationimage showing zones of different porosities in more detail.

To validate the programmed etch structure of the pSi RFs, cross-sectional FE-SEM images of sample RF S2 were recorded. As shown in Figure 5a,b, the rugate structure consisting of a sinusoidal porosity gradient with smooth interfaces in the direction perpendicular to the plane of the filter is visible. The higher and lower porosities, corresponding to larger and smaller current densities, can be clearly distinguished as darker and brighter zones. A periodic pattern is maintained over the whole layer, which consists of 40 periods with an overall thickness of ~11.5 μm. It is also important to mention that the top surface of the pSi RFs strongly resemble the mesoporous surface of the pSi S1 shown in Figure 3a.

**Figure 6:**
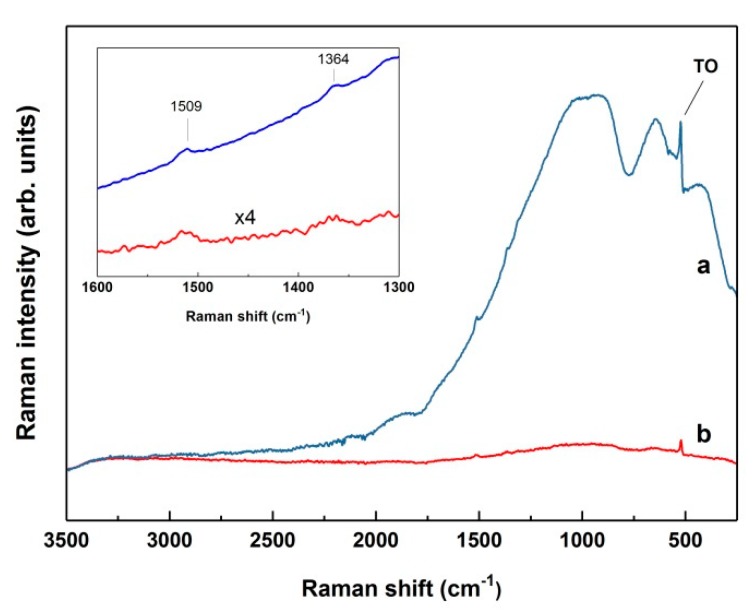
Comparison of FT-Raman spectra of the highest detectable R6G concentration of (10^−2^ M) on non-metallized samples: (**a**) pSi S1, (**b**) RF S2 (both 100 scans, 500 mW). Wavenumber range of 1600–1300 cm^−1^ is magnified in inset. Two weak peaks at 1509 and 1364 cm^−1^ corresponding to the most prominent bands of R6G are visible.

As displayed in Figure 4, the excitation laser wavelength is aligned with the stopband of samples RF S1, RF S2, and RF S2a, hence the high reflection of the incoming light is expected. It is important to mention that no difference was observed for the Raman or SERS intensities of the probe molecules of all three aforementioned substrates, hence only the spectra obtained from sample RF S2 will be presented. Figure 6 presents a comparison of two FT-Raman spectra: Highly concentrated R6G (10^−2^ M) on samples of pSi S1 and RF S2, both on top of a highly doped cSi substrate, prior to Ag deposition. Although both spectra show a Raman TO peak at 520 cm^−1^, it can be concluded that the ~85% reflectance on 1064 nm (visible on Figure 4) of sample RF S2 is enough to successfully quench cSi PL. Also visible are very small peaks corresponding to the most prominent bands of the probe molecule, R6G, exhibiting detection of 10^−2^ M R6G on bare samples of pSi S1 and RF S2, i.e., without Ag coating. The same concentration was also barely detected for CV (not shown). Any Raman signal for concentrations lower than 10^−2^ M of both analytes could not be detected.

### 3.3. SERS on Porous Silicon Photonic Crystals

**Figure 7:**
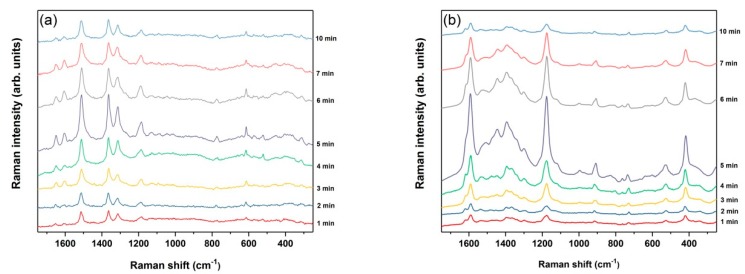
The SERS spectra of 10^−4^ M (**a**) R6G (25 scans, 250 mW) and (**b**) CV (50 scans, 500 mW) molecules deposited on RF S2 samples immersed in 10^−2^ M AgNO_3_ for different times (1 to 10 min from bottom to top). Spectra are offset along the *y*-axis for clarity.

To optimize the immersion plating procedure that adjusts the Ag-coated pSi morphology for maximal SERS enhancement, RF S2 samples were immersed into 10^−2^ M AgNO_3_ for different deposition times (1–10 min) under a fixed solution temperature. This particular Ag salt concentration, according to previous works [8,10,12,14,20,22], was revealed as optimal for the formation of a reproducible and highly effective SERS-active mesoporous silicon because of the fast yet controllable Ag deposition. Due to their well-characterized Raman features and adsorbability onto Ag NPs, we used commonly investigated dye molecules, R6G and CV, as the probe molecules for SERS detection. Figure 7a,b present the comparison of the SERS spectra obtained from the series of Ag-coated RF S2 samples synthesized with different dipping times using 10^−4^ M R6G and CV, respectively. The strongest peak intensities were for both analytes obtained for an immersion duration of exactly 5 min, although the signal is rather strong in the 4–7 min range. For the other immersion times, a significant decrease in the signal intensities can be seen, which is more pronounced in the CV case probably due to the higher laser power used. Referring to Figure 2, it is also apparent that a deposition time of more than 30 min, which quenches the cSi PL on pSi sample S1, would yield a rather small (or none whatsoever) SERS signal, hence the necessity of pSi PhC utilization is further corroborated.

**Figure 8:**
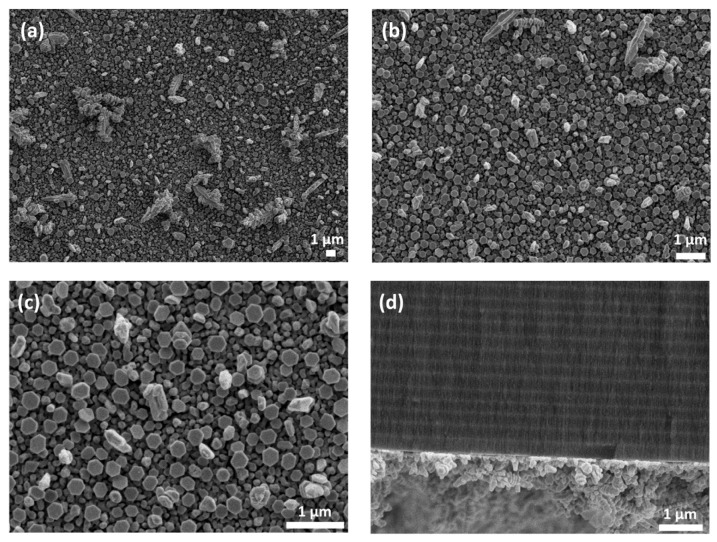
FE-SEM images of Ag NPs, formed on sample RF S2 by immersion plating for 5 min in 10^−2^ M AgNO_3_. (**a**–**c**) Top view micrographs at three magnifications; (**d**) cross-section view.

Since the SERS effects are related to the shape, size, and aggregation of Ag NPs, FE-SEM images of the sample RF S2 immersed in 10^−2^ M AgNO_3_ for 5 min were recorded and examined to reveal the optimal morphology of the Ag-coated pSi RF that produced maximal SERS enhancement. Figure 8a–c show the FE-SEM top-view images of the Ag NP-decorated sample, RF S2, and Figure 8d shows the corresponding cross-section image. Although several dendritic structures already formed by agglomeration can be seen, the 5-min immersion process led to the formation of a large number of randomly deposited, densely packed Ag NPs with a wide size distribution. Moreover, it can be inferred from Figure 8c that a new population of smaller NPs within the gaps between larger ones’ forms, evidencing not only the presence of new nucleation sites for Ag NPs growth, but also a reduction of the gaps between NPs. These narrow gaps might explain the obtained signal enhancement since their presence is known to be essential in obtaining high SERS activity due to the existence of substantially enhanced local EM fields between them [57].

**Figure 9:**
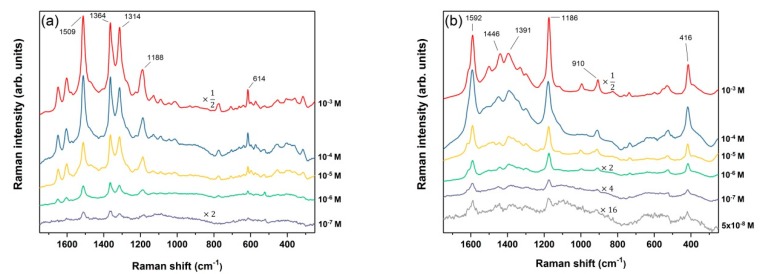
The SERS spectra of (**a**) R6G and (**b**) CV (both 100 scans, 500 mW), deposited on RF S2 samples at different dilutions (higher to lower concentrations from top to bottom). Spectra are offset along the *y*-axis for clarity.

After finding the optimal duration of an immersion process that displayed the highest SERS sensitivity, a series of identical Ag-coated pSi samples RF S2 were soaked in different concentrations of R6G and CV in order to obtain the detection limit. The recorded SERS spectra of the probe molecules with decreasing concentrations are shown in Figure 9a,b. It is clearly visible that the minimum detectable concentration for R6G is 10^−7^ M while for CV we managed to detect a two-times lower concentration of 5 × 10^−8^ M. Although less intensive peaks are not detectable at the lowest concentrations, a few of the most prominent bands of R6G and CV are still well-resolved. They include 614 cm^−1^ (C-C-C ring), 1188 cm^−1^ (C-C stretching), 1314 cm^−1^ (CH deformation), 1364 cm^−1^ and 1509 cm^−1^ (both aromatic C-C stretching) for R6G [58,59]. For CV 416 cm^−1^ (C-C-C out-of-plane bending). 910 cm^−1^ (phenyl ring breathing mode), 1186 cm^−1^ (ring C-H in-plane bending), 1391 cm^−1^ (N-phenyl stretching), 1446 cm^−1^, and 1592 cm^−1^ (both ring C-C stretching) were apparent [12,60]. It is apparent that the positions of these most intense vibrations correlate with the reference peaks, although some small shifts are observed. A possible cause for these shifts might be the adsorption of the dyes with different orientations on the Ag NPs. Evident also are changes in the relative intensities of several SERS bands when comparing ours with reference spectra obtained using visible excitation.

A typical parameter used for estimation of the SERS substrate performance called the enhancement factor is usually calculated [61]. Due to the complex morphology of Ag-coated pSi and the intrinsic difficulty of precisely determining the scattering volume in FT-Raman measurements, the number of molecules contributing to the SERS signal cannot be reliably determined. By virtue of this, the parameter of choice in our investigation used to estimate SERS enhancement was external amplified Raman efficiency (EARE) [62], defined as the ratio of the minimum detectable concentration of the probe molecules obtained on the bare pSi and the one obtained on the SERS substrate. For the SERS substrate, RF S2, the calculated EAREs are 10^5^ for R6G and 5 × 10^5^ for CV. 

The maximum absorptions of R6G and CV are at ~525 and ~590 nm, respectively, hence our excitation wavelength is off-resonant with the analytes’ electronic transitions and no resonance Raman effects, which enhance the SERS signal, are present. Also, the plasmonic resonance of Ag-coated pSi RFs, determined by the size and shape of NPs and their mutual EM interaction, as well as the dielectric function of a pSi substrate, is usually restricted to the visible range [63,64] with only its wings expanding to the NIR region. Therefore, our excitation wavelength is off-resonant with the localized surface plasmon resonance of Ag NPs deposited on pSi RFs. Ergo the obtained SERS enhancement is most likely to be caused by adsorption of molecules onto appropriately structured arrangements of Ag NPs called ‘hot spots’. They include narrow junctions between two or more interacting metal particles, where a spatially confined, intense EM field is present, but also sharp tips of the NPs, where the crowding of the electric field lines exist (lightning rod effect) [65]. Our explanation is in agreement with [66], who concluded that the dye molecules, such as R6G and CV, might preferentially adsorb on hot-spots.

Regarding the issue of reproducibility, since our excitation laser beam with a diameter of ~0.25 mm illuminates a large sample area with randomly adsorbed molecules, which statistically includes many hot spots with different local geometry and hence different enhancement, we did not observe the strong intensity fluctuations notorious for SERS when recording spectra on different spots on samples’ surfaces, although the reproducibility standards proposed in [67] were not fully satisfied.

However, an additional possibility for a small contribution to SERS signal enhancement also exists. As already emphasized, the transmission of light through the PBG is prohibited, hence high back-reflection of the excitation light for sample RF S2, where the PBG is aligned with the laser light, is expected. Thus, the back reflected light might contribute to the enhancement of the local EM field around Ag NPs for pSi RFs and therefore also contribute to the overall SERS enhancement. Moreover, if the wavenumber of the Raman-scattered photons falls within the PBG of a periodic multilayered structure, a backward reflection of photons will result in their constructive interference, leading to further enhancement of Raman signals [68,69]. This principle was also expanded for SERS measurements [70] and, most recently, Zhong et al. [71] reported an almost 4 fold increase in SERS EF by using pSi PhC. Since the primary goal of our PBG was quenching the cSi PL, laser excitation light was aligned with the rather narrow stopband of our pSi RF (<200 nm), hence only low-frequency Raman modes might be enhanced. Increasing the bandwidth of the pSi RF stopband, attainable either by varying the spatial frequency (‘chirping”) [53] or by superposition of sine functions [72], in order to quantify the PBG effect contribution to the SERS enhancement, is the scope of our future work. 

## 4. Conclusions

In conclusion, silver-coated porous silicon photonic crystals as SERS substrates were developed for near-infrared excitation (1064 nm), which is important for the Raman detection and study of fragile biomolecules. Porous silicon photonic crystals with high reflectance at the excitation wavelength were produced to quench the underlying crystalline silicon substrate band gap photoluminescence. The procedure for the formation of the nanostructured silver deposits with an appropriate morphology on the porous layer was optimized, yielding the most pronounced SERS activity for porous silicon rugate filters immersed for 5 min in 10^−2^ M AgNO_3_. The results show high SERS-sensitivity with a concentration detection limit of 10^−7^ and 5 × 10^−8^ M of the typical probe molecules, Rhodamine 6G and Crystal violet, which is a more than 5 orders of magnitude lower concentration than detectable on bare porous silicon. Due to the non-resonant matching of the excitation wavelength with the localized surface plasmon resonance of the silver nanoparticles deposited on the mesoporous silicon, the observed Raman signal enhancement is explained to primarily be the result of the existence of hot-spots on the sample surface. 

## Figures and Tables

**Table 1:** Preparation conditions for all used samples.

Sample	J (mA/cm^2^)	Period (s)	Cycles
pSi S1	50.5	n/a	n/a
RF S1	1–100	4.6	40
RF S2	1–100	4.7	40
RF S2a	1–100	4.7	80
RF S3	1–100	4.8	40
RF S4	1–100	4.9	40
